# Electrocardiographic and echocardiographic abnormalities in urban African people living with HIV in South Africa

**DOI:** 10.1371/journal.pone.0244742

**Published:** 2021-02-02

**Authors:** Geert V. T. Roozen, Ruchika Meel, Joyce Peper, William D. F. Venter, Roos E. Barth, Diederick E. Grobbee, Kerstin Klipstein-Grobusch, Alinda G. Vos

**Affiliations:** 1 Julius Global Health, Julius Center for Health Sciences and Primary Care, University Medical Center Utrecht, Utrecht University, Utrecht, The Netherlands; 2 Division of Cardiology, Department of Internal Medicine, Chris Hani Baragwanath Hospital and University of the Witwatersrand, Johannesburg, South Africa; 3 Department of Cardiology, St. Antonius Hospital, Nieuwegein, The Netherlands; 4 Ezintsha, Wits Reproductive Health and HIV Institute, Faculty of Health Sciences, University of Witwatersrand, Johannesburg, South Africa; 5 Department of Infectious Disease, University Medical Center Utrecht, Utrecht University, Utrecht, The Netherlands; 6 Division of Epidemiology and Biostatistics, School of Public Health, Faculty of Health Sciences, University of the Witwatersrand, Johannesburg, South Africa; Ohio State University, UNITED STATES

## Abstract

**Background:**

Studies from high income countries report that HIV-positive people have an impaired systolic and diastolic cardiac function compared to HIV-negative people. It is unclear if results can be translated directly to the Sub-Saharan Africa context. This study assesses electro- and echocardiographic characteristics in an urban African population, comparing HIV-positive people (treated and not yet treated) with HIV-negative controls.

**Methods:**

We conducted a cross-sectional study in Johannesburg, South Africa. We enrolled HIV-positive participants from three randomized controlled trials that had recruited participants from routine HIV testing programs. HIV-negative controls were recruited from the community. Data were collected on demographics, cardiovascular risk factors, medical history and electrocardiographic and echocardiographic characteristics.

**Results:**

In total, 394 HIV-positive participants and 153 controls were enrolled. The mean age of HIV-positive participants was 40±9 years (controls: 35±10 years), and 34% were male (controls: 50%). Of HIV-positive participants 36% were overweight or obese (controls: 44%), 23% had hypertension (controls: 28%) and 12% were current smoker (controls: 37%). Median time since HIV diagnosis was 6.0 years (IQR 2.3–10.0) and median treatment duration was 4.0 years (IQR 0.0–8.0), 50% had undetectable viral load. The frequency of anatomical cardiac abnormalities was low and did not differ between people with and without HIV. We observed no relation between HIV or anti-retroviral therapy (ART) and systolic or diastolic heart function. There was an association between ART use and corrected QT interval: +11.8 ms compared to HIV-negative controls (p<0.01) and +18.9 ms compared to ART-naïve participants (p = 0.01). We also observed a higher left ventricular mass index in participants on ART (+7.8 g/m^2^, p<0.01), but this association disappeared after adjusting for CD4 cell count, viral load and HIV-duration.

**Conclusion:**

The low number of major cardiac abnormalities in this relatively young, well managed urban African HIV-positive population is reassuring. The increase in corrected QT interval and left ventricular mass may contribute to higher cardiac mortality and morbidity in people living with HIV in the long term.

## Introduction

Non-communicable diseases, such as cardiovascular disease (CVD), have emerged as a major cause of morbidity and mortality in the aging human immunodeficiency virus (HIV) positive population [1]. People living with HIV (PLHIV) have a higher prevalence of cardiac disease, such as myocardial infarction, as compared to HIV-negative people [2–4]. Although the exact mechanisms are still unclear, the increased CVD risk in PLHIV seems to have a multifactorial aetiology where HIV itself, immune activation, ART and conventional cardiovascular risk factors all play a part [5–7]. Furthermore, HIV-infection is associated with multiple cardiac disorders, like pericardial effusions, arrhythmias, HIV-related heart failure and HIV-associated pulmonary hypertension (PAH) [8].

A recent systematic review consisting of studies from Europe and the United States on the effect of HIV on cardiac structure and function in the era of ART, reported that PLHIV had an impaired systolic and diastolic function, and a higher left ventricular (LV) mass (LVM) index, than HIV-negative people [9]. This may predict a higher risk of heart failure and long-term mortality for PLHIV compared to the HIV-negative population [10–12]. It is unclear if results from studies conducted in high-income settings can be translated directly to PLHIV in Sub-Saharan Africa (SSA), where approximately 70% of the world’s HIV-positive population resides [13]. In Europe and the United States, the majority of PLHIV consist of white men who have sex with men and intravenous drug users [14], who have a higher cardiovascular risk than the general population [15, 16]. In SSA, the majority of PLHIV are black Africans from the general population, with a higher proportion of females than males being HIV-positive [14].

This study aims to assess the relation between HIV and electrocardiographic and echocardiographic characteristics in an urban African population. Furthermore, the association between ART and cardiac structure and function will be evaluated by comparing PLHIV on ART to PLHIV not yet on ART.

## Materials and methods

Between July 2016 and November 2017, we conducted a cross-sectional study in Charlotte Maxeke Johannesburg Academic Hospital, Johannesburg, South-Africa. We included both HIV-positive participants as well as HIV-negative participants. All participants were aged 18 years and over. HIV-positive participants were recruited from three completed or ongoing randomized controlled trials (RCTs) comparing different first-and second line ART regimens. Participants in these RCTs were recruited from public HIV testing sites and HIV clinics in the inner city of Johannesburg. Exclusion that applied to these RCTs were, amongst others, abnormal kidney- and liver function, tuberculosis co-infection, pregnancy and hepatitis B co-infection. Details of the individual studies are referenced [17–19].

We divided HIV-positive participants into three groups, according to which RCT they were recruited from. The first group consisted of newly diagnosed HIV-positive participants without previous exposure to ART. Participants were recruited from an ongoing RCT comparing two new first-line ART regimens with the current standard of care first-line ART (trial WHRI060) [17]. Participants were eligible for inclusion in our study between the moment of enrolment in WRHI060 up to 36 weeks thereafter.

The second group consisted of participants on first-line ART. They were recruited from a RCT comparing two first-line ART regimens that had been completed in 2016 [18]. At the moment of inclusion in our study, participants were on a first-line tenofovir containing regimen for at least 2.5 years.

The third group consisted of participants on second-line ART. They were recruited from an open label randomized study to demonstrate non-inferiority of a low-dose boosted darunavir regimen compared to a boosted lopinavir-based regimen [19]. Inclusion criteria for this trial was second-line ART-use for at least 48 weeks. Participants of this trial were approached for enrolment in our study during the baseline visit or one of the follow-up visits.

**Fig 1:**
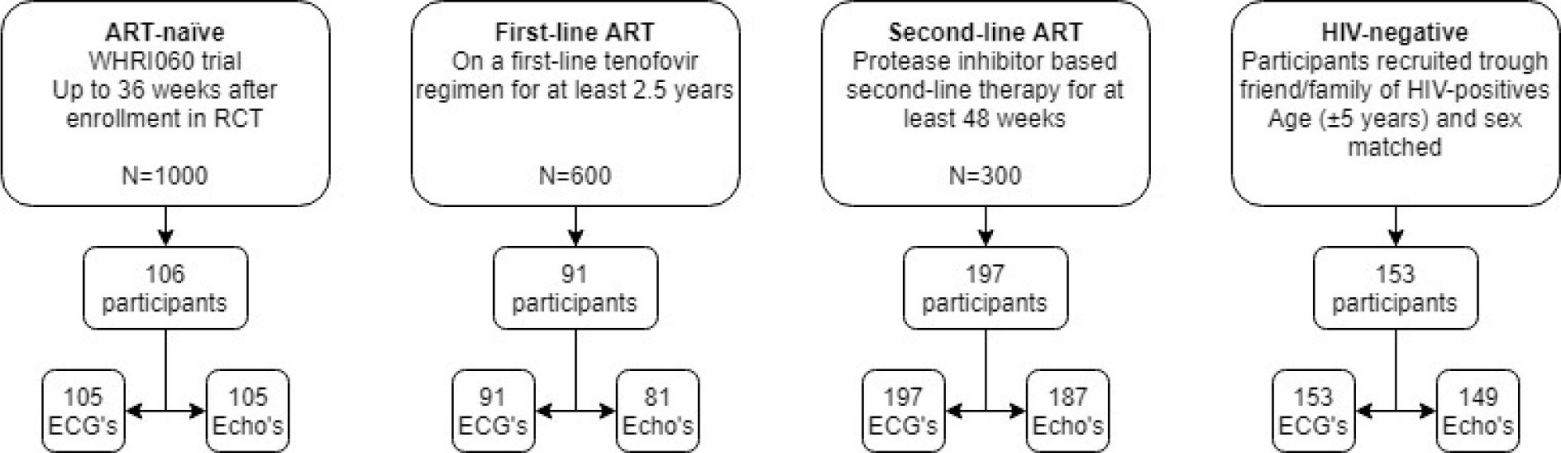
Participant recruitment.

The fourth group consisted of HIV-negative participants. They were recruited through HIV-positive participants to ensure they had the same socio-economic characteristics. HIV-positive participants were invited to refer a family member or friend of the same age (±5 years) and sex without a known seropositive HIV-status. These participants underwent HIV testing upon enrolment according to South African Department of Health HIV testing guideline [20]. Participants who tested HIV-negative, were included in the HIV-negative group. If participants had a positive HIV test results, they were assigned to one of the other groups depending on their ART-status (i.e. naïve, on first-line or on second-line treatment). Participants that tested HIV-positive and were not on ART were referred to a local clinic to initiate treatment. Fig 1 shows a flow diagram of participant recruitment.

Study approval was obtained from the Medical Human Research Ethics Committee of the University of Witwatersrand, Johannesburg, South Africa (M160130). All participants provided written informed consent prior to enrolment.

Data was collected on demographics, life-style, conventional cardiovascular risk factors, medical history and pharmacologic therapy. Information on cardiovascular risk factors was collected using a modified version of WHO-STEPs instrument [21]. Information on physical activity was assessed using the International Physical Activity Questionnaire and accordingly categorised in low, intermediate and high physical activity [22]. Students, retirees, disabled and volunteers were considered unemployed. Participants who stated to have quit smoking less than one month ago were considered current smokers. Pack-years were calculated for current smokers only. Twenty cigarettes, cigars or pipes smoked per day for one year was considered one pack-year. A positive family history of CVD was defined as a history of stroke and/or heart attack of a first-degree family member (parent or sibling) before the age of 60. A self-reported history of stroke, myocardial infarction or angina pectoris was considered a positive history for CVD.

A physical examination included measurement of height, weight, waist circumference, blood pressure and heart rate. Blood pressure was measured with an electronic device in a seated position after a five minutes’ rest at the left arm and the right arm, and repeated at the arm with the highest value. The average of the second and third measurement was reported. Waist circumference was measured halfway between the lower rib and the iliac crest during expiration, in standing position. For the HIV-negative controls, blood was drawn for the analysis of total cholesterol, high-density lipoprotein (HDL) cholesterol, low-density lipoprotein (LDL) cholesterol, triglycerides, random glucose, and for HIV viral load and CD4 cell count for HIV-positive participants. For the HIV-positive participants’ laboratory data of the RCT visit closest to the visit at our study site was used.

Hypertension, abdominal obesity, dyslipidaemia and metabolic syndrome was defined according to the National Cholesterol Education Program, Adult Treatment Panel III (NCEP ATP III) [23]. Accordingly, hypertension was defined as systolic blood pressure (SBP) of >130 mmHg, diastolic blood pressure (DBP) of >85 mmHg or use of antihypertensive medication. Abdominal obesity was defined as a waist circumference ≥102 cm for men and ≥88 cm for women. Diabetes mellitus was defined as random glucose of >11 mmol/L or the use of blood glucose lowering medication. Dyslipidaemia was defined as elevated triglycerides (≥1.7 mmol/L) and/or reduced HDL cholesterol (<1.0 mmol/L for men and <1.3 mmol/L for women). Metabolic syndrome was defined as ≥ 3 out of: diabetes, hypertension, elevated triglycerides, lowered HDL cholesterol or abdominal obesity.

For all participants the Framingham 10-years CVD risk score [24], and the 5-years CVD risk score from the Data-collection on Adverse Effects of Anti-HIV Drugs Study (D:A:D) [25] has been calculated.

A standard 12-lead electrocardiogram (ECG) was recorded (using a laptop-based ECG device, SE-1515 DP12, EDAN) according to standardized procedure. All ECGs were visually assessed by a physician for atrial fibrillation, atrial flutter, atrioventricular block, bundle branch blocks and intraventricular conduction delays (IVCD). An experienced cardiologist was consulted in cases of any doubts about the findings. Heart rate, PR interval, corrected QT interval (QTc) (according to Bazett’s formula), and R and S-wave were calculated automatically by the ECG software. Left ventricular hypertrophy (LVH) according to the Solokow-Lyon Voltage was defined as SV1 + RV5 ≥35 mV. LVH according to the Cornell Voltage was defined as RaVL +SV3 ≥28 mV for men and ≥20 mV for women. LVH according to the Cornell Product was defined as (RaVL + SV3) x QRS duration ≥2440 mmxms [26].

Transthoracic echocardiography was performed using the Siemens Acuson P500 with a 4–2 MHz phased array transducer for cardiac imaging. Dimensions, volumes and other echocardiographic parameters were measured offline according to the recommendations of the American Society of Echocardiography by two experienced ultrasonographers who were blinded for the HIV-status of the participant [27]. Cardiac chamber dimensions and volumes were indexed by body surface area, calculated according to the Mosteller formula [28]. Left atrial and left ventricular volumes were calculated using the biplane Simpsons method. LVM was calculated using the intraventricular septal thickness (IVS), LV end-diastolic diameter (LVED) and the left ventricular posterior wall thickness (LVPW) with the following formula: LVM = 0.8 x 1.04 x [(IVS + LVED + LVPW)3—LVED^3^] + 0.6g. LVH was defined as a LVM index >95 g/m^2^ for women and >115 g/m^2^ for men. Relative wall thickness (RWT) was calculated with the following formula: RWT = 2 x LVPW / LVED. Geometry of the left ventricle (LV) was categorised as follows: normal (defined as no LVH and RWT ≤0.42), concentric remodelling (defined as no LVH and RWT >0.42), eccentric hypertrophy (defined as LVH and RWT ≤0.42), concentric hypertrophy (defined as LVH and RWT >0.42) [27]. Diastolic function was assessed using the ratio of mitral inflow in early diastole and atrial contraction (E/A ratio), mitral inflow deceleration time (DT) and left atrium (LA) volume index. Diastolic dysfunction was defined as: E/A ratio <0.8 and DT >280 ms or LA volume index >34 mL/m^2^ [29]. PAH was defined as a pulmonary artery pressure >35 mmHg.

### Statistical analysis

Continuous parametric variables were reported as means with standard deviations (SD), continuous non-parametric variables as median with interquartile range (IQR). Categorical variables were reported as frequency counts with percentages. For the comparison of means, an analysis of variance (ANOVA) was used. For categorical variables, a chi-squared test was used. A Fischer’s exact test was used when criteria for a chi-square were not met.

For the comparison of means, and analysis of co-variance (ANCOVA) was used. For categorical variables with a binary outcome, a binary logistic regression was used. For categorical variables with a multinominal outcome, a multinominal logistic regression was used. All analyses were adjusted for age and sex. Multivariable linear regression analysis with dummy coding was used to obtain p-values for every ART group individually (supplements).

To assess the relation between HIV and ART and QTc, multivariable linear regression models with dummy coding for ART-status were performed, using the HIV-negative group as reference group. Three models were created. Model 1 is adjusted for age and sex, model 2 is additionally adjusted for conventional cardiovascular risk factors (ever smoked, body mass index (BMI), systolic blood pressure, HDL cholesterol, LDL cholesterol, triglycerides, and glucose) that had a relevant association (p<0.2) with the dependent variable in univariable analysis. Model 3 is the same as Model 2, but only includes the HIV-positive group using the ART-naïve group as reference group. Model 3 is additionally adjusted for HIV-related factors (CD4 cell count, log_10_ viral load, and HIV-duration) with a relevant association (p<0.2) with the dependant variable in univariable analysis.

In a similar way, the relation between HIV and ART and EF, LVM index, E/A ratio, and LA volume index was assessed. Here, the group on first-line and second-line ART were combined to form the ‘On ART group’, due to the low number of participants on first-line ART with complete echocardiograms (n = 49).

A two-sided p-value <0.05 was considered statistically significant. All statistical analyses were performed using IBM SPSS Statistics software, version 25 (IBM, Armonk, New York, United States).

## Results

A total of 547 participants were recruited. Socio-demographic, cardiovascular and HIV-related characteristics of the study population are shown in Table 1. The HIV-negative group included 153 participants (35±11 years old, 50% male), the ART-naïve group consisted of 106 participants (36±18 years old, 38% male), the group on first-line ART of 91 participants (37±7 years old, 40% male), and the group on second-line ART of 197 participants (43±8 years old, 27% male). The majority of participants were moderately to highly physically active and did not smoke. Hypertension and overweight/obesity was frequently observed in all groups, although less often in the ART-naïve group.

**Table 1:** Socio-demographic, cardiovascular and HIV related characteristics of study population.

	HIV-negative		ART-naïve	1^st^ line ART	2^nd^ line ART	p
	(n = 153)		(n = 106)	(n = 91)	(n = 197)	
**Demographics, n (%)**						
Male sex	77 (50.3)		40 (37.7)	36 (39.6)	54 (27.4)	<0.01
Age, mean (SD), years	35.1 (10.5)		35.5 (8.4)	37.0 (6.6)	43.4 (8.0)	<0.001
*Age in categories*, *years*						<0.001
18–29	57 (37.3)		40 (37.7)	8 (8.7)	5 (2.5)	
30–49	80 (52.3)		60 (56.6)	79 (86.8)	152 (77.2)	
≥50	16 (10.5)		6 (5.7)	4 (4.4)	40 (20.3)	
Partnership status: single	119 (78.3)		87 (82.9)	53 (58.9)	111 (56.3)	<0.001
No or only primary education	12 (7.9)		13 (12.5)	10 (11.1)	27 (13.8)	0.37
Employment status: unemployed	102 (67.1)		50 (47.6)	14 (15.6)	65 (33.0)	<0.001
**Lifestyle, n (%)**						
*Physical activity*						0.47
Low	76 (49.7)		47 (44.8)	32 (35.2)	86 (43.7)	
Moderate	57 (37.3)		42 (40.0)	40 (44.0)	79 (40.1)	
High	20 (13.1)		16 (15.2)	19 (20.7)	32 (16.2)	
*Smoking*						<0.001
Current smoker	56 (36.6)		30 (28.3)	11 (12.1)	17 (8.6)	
Pack-years, median [IQR], years	3.5 [2.4–6.2]		5.8 [1.2–9.9]	2.5 [1.4–5.6]	4.0 [1.5–6.4]	0.38
Previous smoker	6 (3.9)		5 (4.7)	8 (8.8)	14 (7.1)	
Never smoked	91 (59.5)		71 (67.0)	72 (79.1)	166 (84.3)	
Heavy alcohol user	16 (10.5)		4 (3.8)	3 (3.3)	0 (0.0)	<0.001
*Chronic medication use*						
Anti-hypertensive	6 (3.9)		6 (5.7)	3 (3.3)	20 (10.2)	0.05
Anti-diabetic	0 (0.0)		1 (0.9)	0 (0.0)	5 (2.5)	0.10
Lipid lowering	0 (0.0)		0 (0.0)	0 (0.0)	10 (5.1)	<0.001
**HIV-related factors, median [IQR]**						
Known HIV-duration, years	-	0.0 [0.0–0.1]		4.0 [3.2–6.1]	9.0 [7.0–12.3]	<0.001
ART-duration, years	-	0.0 [0.0–0.0]		3.3 [3.0–4.0]	8.0 [6.0–10.5]	<0.001
2^nd^ line ART-duration years	-	-		-	1.0 [0.3–4.0]	N/A
Last CD4 count, cells/mm^3^	-	281 [194–401]		411 [276–576]	619 [430–797]	<0.001
Last CD4 count, n (%), <200 cells/mm^3^	-	21 (27.3)		6 (7.2)	7 (3.7)	<0.001
Last viral load, copies/mL	-	13735 [2082–46539]		39 [39–39]	1 [1–1]	<0.001
*Last viral load*, *categorized*, *n (%)*, *copies/mL*						
<50	-	13 (12.6)		79 (90.8)	180 (95.2)	
50–1000	-	10 (9.7)		3 (3.4)	4 (2.1)	
>1000	-	80 (77.7)		5 (5.7)	5 (2.6)	
**Anthropometric measurements, n (%)**						
BMI, mean (SD), kg/m^2^	26.0 (6.5)		24.6 (5.4)	25.7 (5.5)	27.9 (6.5)	<0.001
*BMI in categories*, *kg/m*^*2*^						<0.01
Underweight; <18.5	8 (5.2)		7 (6.6)	3 (3.3)	3 (1.5)	
Normal; 18.5–25	77 (50.3)		58 (54.7)	47 (51.6)	77 (39.1)	
Overweight; >25–30	34 (22.2)		26 (24.5)	19 (20.9)	50 (25.4)	
Obesity; >30	34 (22.2)		15 (14.2)	22 (24.2)	67 (34.0)	
Abdominal obesity	59 (38.6)		38 (36.2)	31 (34.1)	110 (55.8)	<0.001
**Cardiovascular measurements, mean (SD)**						
Systolic blood pressure, mmHg	124 (18)		119 (15)	124 (17)	122 (19)	0.18
Diastolic blood pressure, mmHg	78 (13)		75 (9)	81 (10)	78 (12)	<0.01
Heart rate, beats/minute	70 (11)		72 (10)	74 (12)	68 (11)	<0.01
**Biochemical measurements, mean (SD)**						
Total cholesterol, mmol/L	4.1 (0.9)		3.9 (0.8)	4.4 (1.1)	4.8 (0.8)	<0.001
HDL cholesterol, mmol/L	1.3 (0.5)		1.2 (0.5)	1.4 (0.4)	1.4 (0.4)	0.01
LDL cholesterol, mmol/L	2.2 (0.7)		2.3 (0.7)	2.5 (0.9)	3.1 (0.8)	<0.001
Triglycerides, mmol/L	1.1 (0.6)		1.0 (0.4)	1.3 (0.8)	1.4 (0.9)	<0.001
Random glucose, mmol/L	4.9 (1.0)		4.5 (0.9)	5.0 (0.9)	4.8 (1.6)	0.03
**Cardiovascular risk factors, n (%)**						
Hypertension	42 (27.5)		20 (19.0)	36 (40.0)	65 (33.0)	0.01
Diabetes	0 (0.0)		1 (1.0)	0 (0.0)	6 (3.3)	0.05
Dyslipidaemia	54 (37.0)		55 (55.0)	32 (38.1)	95 (49.7)	0.01
Metabolic syndrome	11 (7.6)		10 (10.0)	11 (13.1)	43 (22.8)	<0.001
Positive family history	7 (4.6)		2 (1.9)	4 (4.4)	21 (10.7)	0.01
History of cardiovascular disease	3 (2.0)		0 (0.0)	3 (3.3)	5 (2.6)	0.32
**CVD prediction models, median [IQR]**						
Framingham, 10-year CVD risk, %	1.8 [0.9–3.4]		1.6 [0.9–2.8]	2.0 [1.3–3.0]	3.0 [1.5–5.3]	<0.001
D:A:D, 5-year CVD risk, %	-		0.3 [0.2–0.6]	0.5 [0.3–0.8]	1.0 [0.6–2.0]	<0.001

Abbreviations: ART = antiretroviral therapy; BMI = body mass index; CVD = cardiovascular disease; D:A:D: = Data Collection of Adverse Events on Anti-HIV Drugs; HDL = high-density lipoprotein; HIV = human immune deficiency virus; IQR = inter quartile ranges; LDL = low-density lipoprotein; SD = standard deviation.

The group on first-line ART had a median duration of known HIV-infection of four years, with an approximate median treatment duration of three years. The group on second-line ART had a median HIV-duration of nine years, with median of eight years of treatment (of which one year of second-line treatment). The majority of the participants on first and second-line ART had an undetectable viral load (91% and 95% respectively).

ECG was available for 545 of 547 participants (99.6%). Rhythm, blocks and IVCD could not be assessed for 10 of 545 ECGs (2%) because of missing files. These incomplete ECGs were spread across groups as follows: 3 in the HIV-negative group (2%), 1 in the ART-naïve group (1%), 2 in the group on first-line ART (2%), and 4 in the group on second-line ART (2%). ECG characteristics are shown in Table 2 and S1 Table. Rhythm, rate and conduction system abnormality did not differ significantly between groups when adjusting for age and sex. Compared to the HIV-negative controls, the group on 1^st^ line ART had a significantly longer QTc (402±27 ms vs. 416±26 ms respectively, p<0.01)), when adjusting for age and sex (see supplements for individual p-values). The group on second line ART had more cases with intraventricular conduction delay than the other groups (p <0.01) and a higher percentage of first degree AV-block, although this was not significant.

**Table 2:** ECG.

ECG finding, n (%)	HIV-negative	ART-naïve	1^st^ line ART	2^nd^ line ART	p^a^
	(n = 153)	(n = 106)	(n = 91)	(n = 195)	
*Rhythm*					0.99
Sinus rhythm	148 (98.7)	104 (99.0)	89 (100)	191 (100)	
Ectopic rhythm	2 (1.3)	1 (1.0)	0 (0.0)	0 (0.0)	
Sinus tachycardia (>100 bpm)	3 (2.0)	1 (0.9)	0 (0.0)	0 (0.0)	0.98
Sinus bradycardia (<60 bpm)	48 (31.4)	27 (25.5)	27 (29.7)	71 (36.4)	<0.01
Shortened PR-interval (<120 ms)	13 (8.5)	10 (9.4)	8 (8.8)	7 (3.6)	0.27
QTc, ms, mean (SD)	402 (27)	407 (24)	416 (26)	413 (33)	0.02
QTc prolongation (males: >440 ms, females: >460 ms)	4 (2.6)	1 (0.9)	7 (7.7)	18 (9.2)	0.08
*AV-block*					0.19
First degree	2 (1.3)	2 (1.9)	1 (1.1)	12 (6.3)	
Second degree	1 (0.7)	1 (1.0)	1 (1.1)	0 (0.0)	
Third degree	0 (0.0)	0 (0.0)	0 (0.0)	0 (0.0)	
Intraventricular conduction delay	48 (32.0)	32 (30.5)	8 (9.0)	53 (27.9)	<0.01
*LBBB*					0.93
Incomplete (QRS 100–120 ms)	4 (2.7)	1 (1.0)	2 (2.2)	3 (1.6)	
Complete (QRS >120 ms)	0 (0.0)	0 (0.0)	0 (0.0)	1 (0.5)	
*RBBB*					0.97
Incomplete (QRS 100–120 ms)	0 (0.0)	3 (2.9)	2 (2.2)	7 (3.7)	
Complete (QRS >120 ms)	0 (0.0)	0 (0.0)	0 (0.0)	0 (0.0)	
LVH acc. Solokow-Lyon voltage	38 (24.8)	32 (30.2)	16 (17.6)	29 (14.9)	0.19
LVH acc. Cornell voltage	15 (9.8)	7 (6.6)	7 (7.7)	20 (10.3)	0.56
LVH acc. Cornell product	8 (5.2)	1 (0.9)	1 (1.1)	6 (3.1)	0.28

^a^Adjusted for age and sex.

Abbreviations: AV-block = atrioventricular block; ART = anti-retroviral therapy; ECG = electrocardiogram; HIV = human immune deficiency virus; LBBB = left bundle branch block; LVH = left ventricular hypertrophy; QTc = corrected QT interval; RBBB = right bundle branch block; SD = standard deviation.

Echocardiogram was available for 522 of 547 participants (95%). Dimensions and volumes could not be measured for 50 of 522 (10%) echocardiograms as the scans could only be assessed visually because of a technical error. These incomplete echocardiograms were spread across groups as follows: 6 in the HIV-negative group (4%), 5 in the ART-naïve group (5%), 32 in the group on first-line ART (40%), and 7 in the group on second-line ART (4%).

Echocardiographic characteristics are shown in Table 3 and S2 Table. There were no significant differences in LV, LA or right ventricular dilation, diastolic dysfunction, right ventricular function, valve dysfunction or pericardial effusion between groups when adjusting for age and sex. None of the participants had PAH. Compared to the HIV-negative controls, the group on first-line ART had significantly more participants with concentric remodelling (20% vs. 45% respectively, p = 0.03), and the group on second-line ART had significantly more participants with eccentric hypertrophy (5% vs. 13% respectively, p = 0.03) (see supplements for individual p-values). The group on second line ART were more likely to have left ventricular dilatation, LVH and diastolic dysfunction as measured from E:A ratio, although differences were non-significant.

**Table 3:** Echocardiography.

	HIV-negative	ART-naïve	1^st^ line ART	2^nd^ line ART	p^a^
	(n = 149)	(n = 105)	(n = 81)	(n = 187)	
**Left Ventricle, mean (SD)**					
LVED index, mm/m^2^	26 (3)	26 (3)	26 (3)	26 (3)	0.29
*LV dilatation*, *n (%)*					0.13
Normal (≤31 mm/m^2^)	138 (96.5)	99 (99.0)	49 (100)	173 (96.1)	
Mild (32–34 mm/m^2^)	4 (2.8)	1 (1.0)	0 (0.0)	5 (2.8)	
Moderate—severe (≥35 mm/m^2^)	1 (0.7)	0 (0.0)	0 (0.0)	2 (1.1)	
LVESD index, mm/m^2^	16 (3)	17 (3)	15 (4)	16 (3)	0.01
LV EDV index, mL/m^2^	57 (15)	58 (15)	56 (11)	53 (15)	0.51
LV ESV index, mL/m^2^	24 (8)	25 (9)	24 (7)	22 (8)	0.05
LVM index, g/m^2^	76 (21)	73 (18)	80 (19)	83 (21)	0.03
LVH, n (%)	10 (7.0)	6 (6.1)	6 (12.2)	33 (18.3)	0.09
Geometry, n (%)					0.03
Normal	105 (73.4)	79 (79.0)	26 (53.1)	94 (52.2)	
Concentric remodelling	28 (19.6)	15 (15.0)	17 (34.7)	53 (29.4)	
Eccentric hypertrophy	7 (4.9)	5 (5.0)	5 (10.2)	23 (12.8)	
Concentric hypertrophy	3 (2.1)	1 (1.0)	1 (2.0)	10 (5.6)	
**Left atrium, mean (SD)**					
LA index, mm/m^2^	18 (2)	19 (2)	19 (2)	19 (2)	0.09
LA volume index, mL/m^2^	18 (6)	19 (5)	17 (4)	18 (6)	0.15
LA dilatation (>34 mL/m^2^), n (%)	2 (1.4)	0 (0.0)	0 (0.0)	3 (1.7)	0.99
Collapse IVC >50%, n (%)	143 (100)	101 (98.1)	78 (98.7)	176 (100)	0.98
**Systolic and diastolic function, mean (SD)**					
Simpsons EF, %	59 (6)	58 (6)	57 (10)	60 (6)	0.06
Depressed EF (<55%), n (%)	35 (26.1)	27 (27.8)	20 (43.5)	36 (20.7)	0.04
Mitral inflow E/A ratio	1.62 (0.48)	1.63 (0.43)	1.48 (0.31)	1.40 (0.39)	0.74
Mitral inflow deceleration time, ms	188 (45)	180 (40)	188 (36)	191 (38)	0.79
Diastolic dysfunction, n (%)	6 (4.2)	1 (1.0)	0 (0.0)	11 (6.3)	0.81
**Right ventricle, mean (SD)**					
RV base index, mm/m^2^	20 (3)	21 (3)	20 (3)	20 (3)	0.26
*RV dilated (eyeball)*, *n (%)*					0.14
Normal	133 (89.9)	92 (87.6)	74 (92.5)	159 (85.9)	
Mild	14 (9.5)	8 (7.6)	6 (7.5)	25 (13.5)	
Moderate	1 (0.7)	5 (4.8)	0 (0.0)	1 (0.5)	
Decreased systolic RV function (TAPSE <16 mm), n (%)	3 (2.3)	0 (0.0)	0 (0.0)	1 (0.6)	0.31
Pulmonary artery hypertension (PAP >35 mmHg), n (%)	0 (0.0)	0 (0.0)	0 (0.0)	0 (0.0)	N/A
**Valves, mean (SD)**					
*Mitral valve*, n (%)					0.06
Normal	127 (85.2)	82 (78.1)	54 (66.7)	152 (82.2)	
Trivial—mild MR	20 (13.4)	23 (21.9)	25 (30.9)	30 (16.2)	
Moderate–severe MR	0 (0.0)	0 (0.0)	0 (0.0)	1 (0.5)	
Other pathology	2 (1.3)	0 (0.0)	2 (2.5)	2 (1.1)	
*Aortic valve*, n (%)					0.45
Normal	148 (99.3)	105 (100)	79 (98.8)	179 (96.2)	
Trivial—mild AR	1 (0.7)	0 (0.0)	1 (1.2)	6 (3.2)	
Moderate—severe AR	0 (0.0)	0 (0.0)	0 (0.0)	1 (0.5)	
Aortic valve stenosis (PG >20 mmHg), n (%)	0 (0.0)	0 (0.0)	0 (0.0)	0 (0.0)	N/A
*Tricuspid valve*, n (%)					0.77
Normal	115 (77.2)	79 (75.2)	63 (77.8)	142 (77.2)	
Trivial—mild TR	34 (22.7)	26 (24.8)	18 (22.2)	40 (21.7)	
Moderate—severe TR	0 (0.0)	0 (0.0)	0 (0.0)	2 (1.1)	
*Pulmonary valve*, n (%)					0.18
Normal	124 (83.8)	79 (75.2)	62 (76.5)	142 (76.8)	
Trivial—mild PR	24 (16.2)	26 (24.8)	19 (23.5)	43 (23.2)	
Pulmonary valve stenosis (PG >36 mmHg), n (%)	0 (0.0	0 (0.0	0 (0.0	0 (0.0	N/A
**Other, n (%)**					
Pericardial effusion	0 (0.0)	3 (2.9)	2 (2.5)	5 (2.7)	1.00

^a^Adjusted for age and sex.

Abbreviations: AR = aortic valve regurgitation; EDV = end-diastolic volume, E/A = early diastole/atrial contraction; EF = ejection fraction; ESV = end-systolic volume; HIV = human immune deficiency virus; IVC = inferior vena cava; LA = left atrium; LV = left ventricle; LVED = left ventricular end-diastolic diameter; LVESD = left ventricular end-systolic diameter; LVH = left ventricular hypertrophy; LVM = left ventricular mass; MR = mitral valve regurgitation; PG = peak gradient; PR = pulmonary valve regurgitation; RV = right ventricle; RVSP = Right Ventricular Systolic Pressure; SD = standard deviation; TAPSE = tricuspid annular plane excursion; TR = tricuspid valve regurgitation.

Table 4 shows the relation between HIV, ART and QTc. Compared to the HIV-negative controls, the group on first-line ART had longer QTc (+11.8 ms, p<0.01) than the HIV-negative controls, independent of cardiovascular risk factors. An even stronger effect was found when taking the ART-naïve group as reference group, comparing with the group on first-line ART. The group on first-line ART had a longer QTc (+19.9ms, p = 0.01), independent of cardiovascular risk factors and HIV-related factors. Infection with HIV on its own or second-line ART use were not associated with a longer QTc.

**Table 4:** The relation between HIV, ART and QTc.

	HIV-negative	ART-naïve	p	1^st^ line ART	p	2^nd^ line ART	p
**QTc, β (95% CI), ms**	n = 153	n = 106		n = 91		n = 195	
Model 1 (n = 544)	REF	3.1 (-3.7, 9.8)	0.37	**11.6 (4.5, 18.7)**	**<0.01**	3.4 (-2.9, 9.6)	0.29
Model 2 (n = 518)	REF	3.7 (-3.4, 10.8)	0.31	**11.8 (4.3, 19.2)**	**<0.01**	4.3 (-2.5, 11.2)	0.22
Model 3 (n = 342)	-	REF		**18.9 (4.9, 32.8)**	**0.01**	17.5 (-1.4, 36.4)	0.07

Significant results (p<0.05) are shown in bold font.

Model 1 is adjusted for age and sex.

Model 2 is model 1, additionally adjusted for cardiovascular risk factors with a relevant association in univariate analysis (p<0.2).

Model 3 is model 2, additionally adjusted for HIV-related factors with a relevant association in univariate analysis (p<0.2).

Abbreviations: ART = anti-retroviral therapy; CI = confidence interval; HIV = human immune deficiency virus; QTc = corrected QT interval; REF = reference value.

Table 5 shows the relation between HIV, ART and EF, LVM index, E/A ratio, and LA volume index. ART-use was associated with a higher LVM index (+7.8 g/m^2^, p<0.01), independent of cardiovascular risk factors. However, this association disappeared after adjusting for HIV-related factors like CD4 cell count, log_10_ viral load and HIV-duration. We observed no association between HIV or ART and EF, E/A ratio or LA volume index following adjustment.

**Table 5:** The relation of HIV and ART, and EF, LVM index, E/A ratio and LA volume index.

	HIV-negative	ART-naïve	p	On ART	p
**Simpsons EF, β (95% CI), %**	n = 134	n = 98		n = 220	
Model 1 (n = 452)	REF	**-1.8 (-3.5. -0.1)**	**0.04**	-0.5 (-2.0, 1.0)	0.48
Model 2 (n = 440)	REF	-1.6 (-3.3, 0.2)	0.08	-0.5 (-2.1, 1.0)	0.51
Model 3 (n = 279)	-	REF		0.7 (2.9, 4.3)	0.70
**LVM index, β (95% CI), g/m**^**2**^	n = 143	n = 100		n = 229	
Model 1 (n = 472)	REF	-0.7 (-5.8, 4.4)	0.79	**5.5 (1.0, 9.9)**	**0.02**
Model 2 (n = 440)	REF	1.3 (-4.2, 6.7)	0.65	**7.8 (2.7, 12.9)**	**<0.01**
Model 3 (n = 280)	-	REF		0.9 (-10.9, 12.6)	0.89
**E/A ratio, β (95% CI)**	n = 144	n = 102		n = 221	
Model 1 (n = 467)	REF	0.0 (-0.1, 0.1)	0.91	-0.0 (-0.1, 0.1)	0.45
Model 2 (n = 435)	REF	0.0 (-0.1, 0.1)	0.85	-0.0 (-0.1, 0.1)	0.46
Model 3 (n = 274)	-	REF		-0.1 (-0.3, 0.1)	0.34
**LA volume index, β (95% CI), mL/m**^**2**^	n = 138	n = 102		n = 222	
Model 1 (n = 462)	REF	0.8 (-0.7, 2.3)	0.28	-0.7 (-2.0, 0.6)	0.27
Model 2 (n = 462)	REF	1.0 (-0.4, 2.5)	0.16	-0.6 (-1.9, 0.6)	0.33
Model 3 (n = 322)	-	REF		-1.4 (3.1, 0.40)	0.13

The group ‘On ART’ is a combination of the group on first-line ART and second-line ART.

Significant results (p<0.05) are shown in bold font.

Model 1 is adjusted for age and sex.

Model 2 is model 1, additionally adjusted for cardiovascular risk factors with a relevant association in univariate analysis (p<0.2).

Model 3 is model 2, additionally adjusted for HIV-related factors with a relevant association in univariate analysis (p<0.2).

Abbreviations: ART = anti-retroviral therapy; CI = confidence interval; E/A = early diastole/atrial contraction; EF = ejection fraction; HIV = human immune deficiency virus; LA = left atrium; LVM = left ventricular mass; REF = reference value.

## Discussion

In this large, relatively young, urban African group of well-managed PLHIV on ART, not yet on ART and HIV-negative controls, the prevalence of major cardiac conduction disorders was low and there were no clinically relevant arrhythmias. First-line ART use was independently associated with longer QTc. Basic echocardiographic characteristics did not differ between PLHIV and HIV-negative controls. We did not observe an association between ART use and cardiac structure or function. We did, however, observe that participants on ART (first and second-line combined) had a higher LVM index than HIV-negative controls.

HIV-positive participants did not have more major ECG abnormalities than HIV-negative controls, which is a reassuring finding. However, we did observe an independent association between first-line ART and QTc prolongation. Two previous studies also observed longer QTc in PLHIV compared to HIV-negative controls, of +4.5 and +12.5 ms respectively [30, 31]. They did not, however, observe an independent relation between ART and QTc but this might be explained by a low number of ART-naïve participants in these studies and might hence be a lack of statistical power. Efavirenz, the most commonly used non-nucleoside reverse transcriptase inhibitor in first-line ART in South Africa, has been associated with QTc prolongation and might be an explanation of our finding [32]. QTc is a predictor of both overall and CVD mortality and prolonged QTc may predispose individuals to sudden cardiac death [33]. The higher frequency of prolonged QTc we found in participants on first-line ART may be a predictor of cardiac death in this group at a later age. Studies with follow-up are needed to asses if this is truly the case.

The low frequency of dilated cardiomyopathy in our study is reassuring. LV dilatation was present in only a few participants on second-line ART and this was not significantly different from the HIV-negative control group. In addition, we did not find an association between HIV or ART and EF. Prior to the introduction of ART, HIV-related heart failure, a disease that is related to severe immune compromise, was regularly reported in SSA [34, 35]. Our findings support the observation that HIV-related heart failure is hardly seen any more in SSA in the context of well treated HIV [36]. The timely diagnosis of HIV, probably explains why we do not find more heart failure and dilated cardiomyopathy in our HIV-positive, ART-naïve group than in our HIV-negative controls.

Multiple studies, both in high-income countries and SSA, have reported higher rates of diastolic dysfunction in PLHIV compared to HIV-negative people [37–39], and this has been associated with HIV-related factors like nadir CD4 cell count [40]. Diastolic function, as evaluated with E/A ratio and LA volume, did not differ between the groups in our study following adjustment for age and sex. However, our results are limited by the lack of tissue Doppler imaging data and may hence be an underestimation of diastolic dysfunction.

We did not observe PAH in any of the participants. The prevalence of HIV-associated PAH in an high-income setting is 0.5% [41]. Our results might be an underestimation of PAH, because participants did not undergo invasive catheterization to assed pulmonary artery pressure. The ongoing multinational Pan African Pulmonary hypertension Cohort (PAPUCO) study, will give more insight in the epidemiology of HIV-associated PAH in SSA [42].

Another reassuring negative result of this study is the low frequency of valve dysfunction. A study conducted in Germany, among 803 HIV-infected individuals (44±10 years old, 84% male), diagnosed valve dysfunction (mostly regurgitation) in 77% of the participants, of which 5% was clinically relevant [43]. We did not observe such a high frequency of valve problems, and this is surprising given that rheumatic heart disease is still prevalent in Africa. The high percentage of participants with valve problems in the German study possibly reflects the older age of participants (the mean age is approximately 6 years older than in our study) and the high number of intravenous drug users (9%) [43].

Participants on ART (first and second-line combined) had a higher LVM index than HIV-negative controls, independent of cardiovascular risk factors. However, there was no difference in LVM index when comparing between participants on ART and ART-naïve participants, when correcting for HIV-related factors. This indicates that this association depends on HIV-related factors, rather than ART use. This is in accordance to previous studies in both high-income settings and SSA that found a higher LVM index in PLHIV compared to HIV-negative controls [9, 38, 39, 44, 45]. Both higher immune activation and increased atherosclerotic processes have been proposed as a possible cause of the increase in LVM in PLHIV, in addition to conventional risk factors like obesity and hypertension [44, 46]. This would mean that inhibition of viral activity does help in counteracting the process of LVM increase, thus emphasizing the importance of effective ART administration.

A major strength of our study is that it is the largest echocardiographic study conducted in PLHIV and HIV-negative controls in SSA. Another strength is the fact that we enrolled HIV-positive participants on ART and not yet on ART, which enabled us to gain insight into the relation of ART and cardiac function.

The cross-sectional design of our study, however, precluded evaluation of causal relations. Another limitation is the relatively young age of our participants, a relatively short duration since HIV diagnosis and high percentage of participants on ART who were virally suppressed. This may partly explain the relatively low prevalence of pathology that we observed and results might not be generalizable to an older population with less viral control. Third, HIV-positive participants recruited from RCTs were compared to controls from the general population. We acknowledge that this, at first sight, may results in a bias as participants in RCTs differ from the general population. However, HIV-positive participants were recruited at public HIV treatment centres. Study participation provides participants with benefits beyond the normal health care system like scheduled appointments (and so less waiting time), personal attention by dedicated study team members and a reimbursement per visit. As a result, we believe that participation in an RCT was attractive to the majority of participants in the HIV clinics and that participants represent the general HIV-positive population in the inner city of Johannesburg. HIV negative controls were recruited through HIV-positive participants to assure that they represent the same source population. There was a trend for PLHIV on second line ART to have more cardiac abnormalities then the other groups, although not significant. This may be the result of a lack of power as the HIV-negative group is smaller than the HIV-positive group. Finally, the missing echocardiograms for the group on first-line ART precluded the analysis for first- and second-line ART separately.

Our results demonstrate that the frequency of major cardiac abnormalities such as dilated cardiomyopathy, PAH or valvular disease is the same for PLHIV and HIV-negative people in this urban African setting. This is in contrast to what was observed before the introduction of ART, when cardiac abnormalities were frequently seen in PLHIV in SSA [47]. At the same time, both the QTc prolongation and the increase in LVM that we observed in PLHIV on first-line ART may contribute to higher cardiac morbidity and mortality in the long term. Our results emphasize the importance of effective and safe ART administration to ensure healthy aging in PLHIV. Follow-up studies are needed to evaluate if the QTc prolongation and increase in LVM does translate in adverse clinical outcomes.

## Supporting information

S1 Table ECG with individual p-values. ^a^Adjusted for age and sex. Abbreviations: AV-block = atrioventricular block; ART = anti-retroviral therapy; ECG = electrocardiogram; HIV = human immune deficiency virus; LBBB = left bundle branch block; LVH = left ventricular hypertrophy; QTc = corrected QT interval; RBBB = right bundle branch block; REF = reference value; SD = standard deviation.
(DOCX)

S2 Table Echocardiography with individual p-values. ^a^Adjusted for age and sex. Abbreviations: AR = aortic valve regurgitation; EDV = end-diastolic volume, E/A = early diastole/atrial contraction; EF = ejection fraction; ESV = end-systolic volume; HIV = human immune deficiency virus; IVC = inferior vena cava; LA = left atrium; LV = left ventricle; LVED = left ventricular end-diastolic diameter; LVESD = left ventricular end-systolic diameter; LVH = left ventricular hypertrophy; LVM = left ventricular mass; MR = mitral valve regurgitation; PG = peak gradient; PR = pulmonary valve regurgitation; RV = right ventricle; RVSP = Right Ventricular Systolic Pressure; REF = reference value; SD = standard deviation; TAPSE = tricuspid annular plane excursion; TR = tricuspid valve regurgitation.
(DOCX)

S1 Data (XLSX)
