# Supplementary material for: Electrocardiographic and echocardiographic abnormalities in urban African people living with HIV in South Africa
Source: PLoS One. 2021 Feb 2;16(2):e0244742. doi: 10.1371/journal.pone.0244742 (PMC7853516; doi:10.1371/journal.pone.0244742)
Supplement: S1 Table — aAdjusted for age and sex. Abbreviations: AV-block = atrioventricular block; ART = anti-retroviral therapy; ECG = electrocardiogram; HIV = human immune deficiency virus; LBBB = left bundle branch block; LVH = left ventricular hypertrophy; QTc = corrected QT interval; RBBB = right bundle branch block; REF = reference value; SD = standard deviation. (DOCX) [file pone.0244742.s001.docx]

| **S1 Table. ECG with individual p-values** | | | | | | | | |
| --- | --- | --- | --- | --- | --- | --- | --- | --- |
| **ECG finding, n (%)** | **HIV-negative**  **(n=153)** | **p**^a^ | **ART-naïve**  **(n=106)** | **p**^a^ | **1^st^ line ART**  **(n=91)** | **p**^a^ | **2^nd^ line ART**  **(n=195)** | **p**^a^ |
| *Rhythm* |  |  |  |  |  |  |  |  |
| Sinus rhythm | 148 (98.7) | REF | 104 (99.0) | REF | 89 (100) | REF | 191 (100) | REF |
| Ectopic rhythm | 2 (1.3) | REF | 1 (1.0) | 0.90 | 0 (0.0) | 1.00 | 0 (0.0) | 1.00 |
| Sinus tachycardia (>100 bpm) | 3 (2.0) | REF | 1 (0.9) | 0.65 | 0 (0.0) | 1.00 | 0 (0.0) | 1.00 |
| Sinus bradycardia (<60 bpm) | 48 (31.4) | REF | 27 (25.5) | 0.67 | 27 (29.7) | 0.63 | 71 (36.4) | <0.01 |
| Shortened PR-interval (<120 ms) | 13 (8.5) | REF | 10 (9.4) | 0.95 | 8 (8.8) | 0.98 | 7 (3.6) | 0.10 |
| QTc, ms, mean (SD) | 402 (27) | REF | 407 (24) | 0.37 | 416 (26) | <0.01 | 413 (33) | 0.29 |
| QTc prolongation (males: >440 ms, females: >460 ms) | 4 (2.6) | REF | 1 (0.9) | 0.45 | 7 (7.7) | 0.07 | 18 (9.2) | 0.08 |
| *AV-block* |  |  |  |  |  |  |  |  |
| First degree | 2 (1.3) | REF | 2 (1.9) | 0.61 | 1 (1.1) | 0.95 | 12 (6.3) | 0.05 |
| Second degree | 1 (0.7) | REF | 1 (1.0) | 0.63 | 1 (1.1) | 0.57 | 0 (0.0) | 1.00 |
| Third degree | 0 (0.0) | N/A | 0 (0.0) | N/A | 0 (0.0) | N/A | 0 (0.0) | N/A |
| Intraventricular conduction delay | 48 (32.0) | REF | 32 (30.5) | 0.82 | 8 (9.0) | <0.001 | 53 (27.9) | 0.24 |
| *LBBB* |  |  |  |  |  |  |  |  |
| Incomplete (QRS 100 – 120 ms) | 4 (2.7) | REF | 1 (1.0) | 0.43 | 2 (2.2) | 0.96 | 3 (1.6) | 0.67 |
| Complete (QRS >120 ms) | 0 (0.0) | REF | 0 (0.0) | N/A | 0 (0.0) | N/A | 1 (0.5) | 1.00 |
| *RBBB* |  |  |  |  |  |  |  |  |
| Incomplete (QRS 100 – 120 ms) | 0 (0.0) | REF | 3 (2.9) | 1.00 | 2 (2.2) | 1.00 | 7 (3.7) | 1.00 |
| Complete (QRS >120 ms) | 0 (0.0) | N/A | 0 (0.0) | N/A | 0 (0.0) | N/A | 0 (0.0) | N/A |
| LVH acc. Solokow-Lyon voltage | 38 (24.8) | REF | 32 (30.2) | 0.13 | 16 (17.6) | 0.48 | 29 (14.9) | 0.72 |
| LVH acc. Cornell voltage | 15 (9.8) | REF | 7 (6.6) | 0.51 | 7 (7.7) | 0.77 | 20 (10.3) | 0.42 |
| LVH acc. Cornell product | 8 (5.2) | REF | 1 (0.9) | 0.12 | 1 (1.1) | 0.17 | 6 (3.1) | 0.67 |
