# Supplementary material for: Electrocardiographic and echocardiographic abnormalities in urban African people living with HIV in South Africa
Source: PLoS One. 2021 Feb 2;16(2):e0244742. doi: 10.1371/journal.pone.0244742 (PMC7853516; doi:10.1371/journal.pone.0244742)
Supplement: S2 Table — aAdjusted for age and sex. Abbreviations: AR = aortic valve regurgitation; EDV = end-diastolic volume, E/A = early diastole/atrial contraction; EF = ejection fraction; ESV = end-systolic volume; HIV = human immune deficiency virus; IVC = inferior vena cava; LA = left atrium; LV = left ventricle; LVED = left ventricular end-diastolic diameter; LVESD = left ventricular end-systolic diameter; LVH = left ventricular hypertrophy; LVM = left ventricular mass; MR = mitral valve regurgitation; PG = peak gradient; PR = pulmonary valve regurgitation; RV = right ventricle; RVSP = Right Ventricular Systolic Pressure; REF = reference value; SD = standard deviation; TAPSE = tricuspid annular plane excursion; TR = tricuspid valve regurgitation. (DOCX) [file pone.0244742.s002.docx]

| **S2 Table. Echocardiography with individual p-values** | | | | | | | | |
| --- | --- | --- | --- | --- | --- | --- | --- | --- |
|  | **HIV-negative**  **(n=149)** | **p**^a^ | **ART-naïve**  **(n=105)** | **p**^a^ | **1^st^ line ART**  **(n=81)** | **p**^a^ | **2^nd^ line ART**  **(n=187)** | **p**^a^ |
| **Left Ventricle, mean (SD)** |  |  |  |  |  |  |  |  |
| LVED index, mm/m^2^ | 26 (3) | REF | 26 (3) | 0.09 | 26 (3) | 0.86 | 26 (3) | 0.30 |
| *LV dilatation, n (%)* |  |  |  |  |  |  |  |  |
| Normal (≤31 mm/m^2^) | 138 (96.5) | REF | 99 (99.0) | REF | 49 (100) | REF | 173 (96.1) | REF |
| Mild (32-34 mm/m^2^) | 4 (2.8) | REF | 1 (1.0) | 0.43 | 0 (0.0) | 1.00 | 5 (2.8) | 0.72 |
| Moderate - severe (≥35 mm/m^2^) | 1 (0.7) | REF | 0 (0.0) | 1.00 | 0 (0.0) | N/A | 2 (1.1) | 0.05 |
| LVESD index, mm/m^2^ | 16 (3) | REF | 17 (3) | 0.01 | 15 (4) | 0.37 | 16 (3) | 0.38 |
| LV EDV index, mL/m^2^ | 57 (15) | REF | 58 (15) | 0.28 | 56 (11) | 0.94 | 53 (15) | 0.65 |
| LV ESV index, mL/m^2^ | 24 (8) | REF | 25 (9) | 0.04 | 24 (7) | 0.76 | 22 (8) | 0.50 |
| LVM index, g/m^2^ | 76 (21) | REF | 73 (18) | 0.80 | 80 (19) | 0.35 | 83 (21) | 0.01 |
| LVH, n (%) | 10 (7.0) | REF | 6 (6.1) | 0.74 | 6 (12.2) | 0.31 | 33 (18.3) | 0.05 |
| Geometry, n (%) |  |  |  |  |  |  |  |  |
| Normal | 105 (73.4) | REF | 79 (79.0) | REF | 26 (53.1) | REF | 94 (52.2) | REF |
| Concentric remodelling | 28 (19.6) | REF | 15 (15.0) | 0.39 | 17 (34.7) | 0.03 | 53 (29.4) | 0.07 |
| Eccentric hypertrophy | 7 (4.9) | REF | 5 (5.0) | 0.89 | 5 (10.2) | 0.12 | 23 (12.8) | 0.03 |
| Concentric hypertrophy | 3 (2.1) | REF | 1 (1.0) | 0.57 | 1 (2.0) | 0.82 | 10 (5.6) | 0.25 |
| **Left atrium, mean (SD)** |  |  |  |  |  |  |  |  |
| LA index, mm/m^2^ | 18 (2) | REF | 19 (2) | 0.04 | 19 (2) | 0.17 | 19 (2) | 0.03 |
| LA volume index, mL/m^2^ | 18 (6) | REF | 19 (5) | 0.28 | 17 (4) | 0.17 | 18 (6) | 0.46 |
| LA dilatation (>34 mL/m^2^), n (%) | 2 (1.4) | REF | 0 (0.0) | 1.00 | 0 (0.0) | 1.00 | 3 (1.7) | 0.76 |
| Collapse IVC >50%, n (%) | 143 (100) | REF | 101 (98.1) | 1.00 | 78 (98.7) | 1.00 | 176 (100) | 1.00 |
| **Systolic and diastolic function, mean (SD)** | |  |  |  | |  |  |  |
| Simpsons EF, % | 59 (6) | REF | 58 (6) | 0.04 | 57 (10) | 0.07 | 60 (6) | 0.97 |
| Depressed EF (<55%), n (%) | 35 (26.1) | REF | 27 (27.8) | 0.50 | 20 (43.5) | 0.02 | 36 (20.7) | 0.55 |
| Mitral inflow E/A ratio | 1.62 (0.48) | REF | 1.63 (0.43) | 0.91 | 1.48 (0.31) | 0.32 | 1.40 (0.39) | 064 |
| Mitral inflow deceleration time, ms | 188 (45) | REF | 180 (40) | 0.31 | 188 (36) | 0.70 | 191 (38) | 0.57 |
| Diastolic dysfunction, n (%) | 6 (4.2) | REF | 1 (1.0) | 0.33 | 0 (0.0) | 1.00 | 11 (6.3) | 0.78 |
| **Right ventricle, mean (SD)** |  |  |  |  |  |  |  |  |
| RV base index, mm/m^2^ | 20 (3) | REF | 21 (3) | 0.10 | 20 (3) | 0.74 | 20 (3) | 0.25 |
| *RV dilated (eyeball), n (%)* |  |  |  |  |  |  |  |  |
| Normal | 133 (89.9) | REF | 92 (87.6) | REF | 74 (92.5) | REF | 159 (85.9) | REF |
| Mild | 14 (9.5) | REF | 8 (7.6) | 0.86 | 6 (7.5) | 0.59 | 25 (13.5) | 0.67 |
| Moderate | 1 (0.7) | REF | 5 (4.8) | 0.03 | 0 (0.0) | N/A | 1 (0.5) | 0.17 |
| Decreased systolic RV function  (TAPSE <16 mm), n (%) | 3 (2.3) | REF | 0 (0.0) | 1.00 | 0 (0.0) | 1.00 | 1 (0.6) | 0.18 |
| Pulmonary artery hypertension (PAP >35 mmHg), n (%) | 0 (0.0) | N/A | 0 (0.0) | N/A | 0 (0.0) | N/A | 0 (0.0) | N/A |
| **Valves, mean (SD)** |  |  |  |  |  |  |  |  |
| *Mitral valve*, n (%) |  |  |  |  |  |  |  |  |
| Normal | 127 (85.2) | REF | 82 (78.1) | REF | 54 (66.7) | REF | 152 (82.2) | REF |
| Trivial - mild MR | 20 (13.4) | REF | 23 (21.9) | 0.08 | 25 (30.9) | <0.01 | 30 (16.2) | 0.34 |
| Moderate – severe MR | 0 (0.0) | REF | 0 (0.0) | N/A | 0 (0.0) | N/A | 1 (0.5) | 1.00 |
| Other pathology | 2 (1.3) | REF | 0 (0.0) | 1.00 | 2 (2.5) | 0.41 | 2 (1.1) | 0.55 |
| *Aortic valve*, n (%) |  |  |  |  |  |  |  |  |
| Normal | 148 (99.3) | REF | 105 (100) | REF | 79 (98.8) | REF | 179 (96.2) | REF |
| Trivial - mild AR | 1 (0.7) | REF | 0 (0.0) | 1.00 | 1 (1.2) | 0.63 | 6 (3.2) | 0.29 |
| Moderate - severe AR | 0 (0.0) | REF | 0 (0.0) | 1.00 | 0 (0.0) | N/A | 1 (0.5) | 1.00 |
| Aortic valve stenosis  (PG >20 mmHg), n (%) | 0 (0.0 | N/A | 0 (0.0 | N/A | 0 (0.0 | N/A | 0 (0.0 | N/A |
| *Tricuspid valve*, n (%) |  |  |  |  |  |  |  |  |
| Normal | 115 (77.2) | REF | 79 (75.2) | REF | 63 (77.8) | REF | 142 (77.2) | REF |
| Trivial - mild TR | 34 (22.7) | REF | 26 (24.8) | 0.90 | 18 (22.2) | 0.78 | 40 (21.7) | 0.43 |
| Moderate - severe TR | 0 (0.0) | REF | 0 (0.0) | 1.00 | 0 (0.0) | N/A | 2 (1.1) | 1.00 |
| *Pulmonary valve*, n (%) |  |  |  |  |  |  |  |  |
| Normal | 124 (83.8) | REF | 79 (75.2) | REF | 62 (76.5) | REF | 142 (76.8) | REF |
| Trivial - mild PR | 24 (16.2) | REF | 26 (24.8) | 0.10 | 19 (23.5) | 0.14 | 43 (23.2) | 0.04 |
| Pulmonary valve stenosis  (PG >36 mmHg), n (%) | 0 (0.0 | N/A | 0 (0.0 | N/A | 0 (0.0 | N/A | 0 (0.0 | N/A |
| **Other, n (%)** |  |  |  |  |  |  |  |  |
| Pericardial effusion | 0 (0.0) | REF | 3 (2.9) | 1.00 | 2 (2.5) | 1.00 | 5 (2.7) | 1.00 |
